# Bacteriophages shift the focus of the mammalian microbiota

**DOI:** 10.1371/journal.ppat.1007310

**Published:** 2018-10-25

**Authors:** Breck A. Duerkop

**Affiliations:** Department of Immunology and Microbiology, University of Colorado School of Medicine, Aurora, Colorado, United States of America; University of Florida, UNITED STATES

For over three centuries, there has been rising interest in and fascination with the complex consortium of microbes that associate with animals, plants, and other environmental ecosystems, collectively termed the microbiome. Within the last half century, following the advent of culture-independent methods for the surveillance of microbial communities, the microbiome has been studied in unprecedented detail. As a result, it has become increasingly clear that resident microbes are integral to health and disease.

The average human is colonized with over 10 trillion bacteria, which have largely been the focus of host–microbiota research. Aside from bacteria, there are staggering numbers of bacterial viruses known as bacteriophages (phages) that inhabit mammalian microbiotas. Most bacteria harbor one or more phage in their chromosome(s) as quiescent prophage capable of undergoing lytic replication to produce infectious virions. It is not surprising that these viruses are also majority members of the microbiota and may even exceed bacteria in abundance. Phages make up a significant component of diverse microbiomes ranging from mammals, plants, soil, and the ocean [1–4]. These environments are being studied to determine how phages contribute to the assembly, stability, and function of microbial communities. An emerging topic related to the study of phages in host-associated microbiotas is their potential role in mammalian health and disease. Here, I review our current understanding of phages within the context of the mammalian microbiota, discuss how these viruses influence host–microbe interactions, and explore how deciphering phage influence on complex microbial communities could inform the future of microbiota research related to human health and disease.

## Bacteriophages of the microbiota: Composition, evolution, and impact on the ecosystem

Phages are ubiquitous to body surfaces, including the skin, oral cavity, lungs, intestine, and urinary tract [1, 5–10]. The majority have double stranded DNA (dsDNA) genomes and belong to the Caudovirales order of tailed phages [1, 8, 9]. Metagenomic analyses have identified both single stranded DNA (ssDNA) and RNA phages as components of the microbiota [11]. Considering that dsDNA and ssDNA phage metagenomes are more frequently studied, the abundance and diversity of RNA phages are underrepresented and reflect a key knowledge gap of the mammalian microbiota.

We undoubtedly know the most about intestinal phages, and early work characterizing the composition of fecal phage communities from related humans uncovered that unlike fecal bacterial composition, which is conserved among relatives, phage communities were unique but stable [1]. This initial survey of intestinal phages raised questions about whether intestinal phage–bacteria interactions follow a traditional reciprocal predator–prey relationship (i.e., as phage abundances go up, host bacterial abundances drop and vice versa), as observed in other ecosystems such as the ocean. The use of gnotobiotic mice colonized with a defined bacterial community allowed for finer resolution of phage–bacteria interactions over time and revealed that for some phages and their bacterial hosts, reciprocal predator–prey relationships do exist in the intestine and that predatory phages promote the evolution of bacterial phage resistance in response to phage infection [12]. Furthermore, intestinal commensal bacteria carrying prophage DNA were shown to produce infectious virions that facilitate interspecies competition [13]. These studies were instrumental in showing that intestinal bacterial community composition can be dictated by phages, and thus phages likely have a strong influence on shaping the microbiota.

Phage community composition is also driven by external environmental factors. This was shown in the case of perturbed host diet, in which early during the shift from a low-fat to high-fat diet, the intestinal phage community diverged in the absence of bacterial community composition changes [14]. Additionally, the administration of antibiotics was shown to markedly alter both phage and bacterial intestinal communities [15, 16]. Antibiotic treatment induced the transcription of genes associated with the phage lytic cycle and resulted in elevated levels of phage integrase DNA within the phage community, implicating antibiotics in the induction of lytic intestinal prophage replication. During intestinal colonization, increased prophage excision and lytic phage infectivity of *Salmonella enterica* Typhimurium phages are dependent on inflammation. Furthermore, *S*. *enterica* that lack integrated lysogens and are incapable of promoting intestinal inflammation experience reduced prophage acquisition in their genomes [17]. Given these situations, it is clear that both external environmental factors and mammalian host-derived signals encountered by the microbiota are significant drivers of prophage induction and phage community composition, yet the impact of these events on the bacterial community and the mammalian host remain to be elucidated.

The genetic evolution of phages has been linked to their interactions with the microbiota. A recent study reported that phages encountering nonsusceptible bacterial cells in the intestine can first infect and replicate in susceptible hosts in which phage genome diversification occurs, resulting in the propagation of evolved phage progeny that are capable of infecting the previously nonsusceptible bacteria [18]. This phage genome diversification was not observed in axenic mice monocolonized with a single host bacterium or in vitro, highlighting the importance of complex host-associated bacterial communities in promoting phage evolution.

## Bacteriophage interactions with mammalian host barriers and beyond

**Fig 1:**
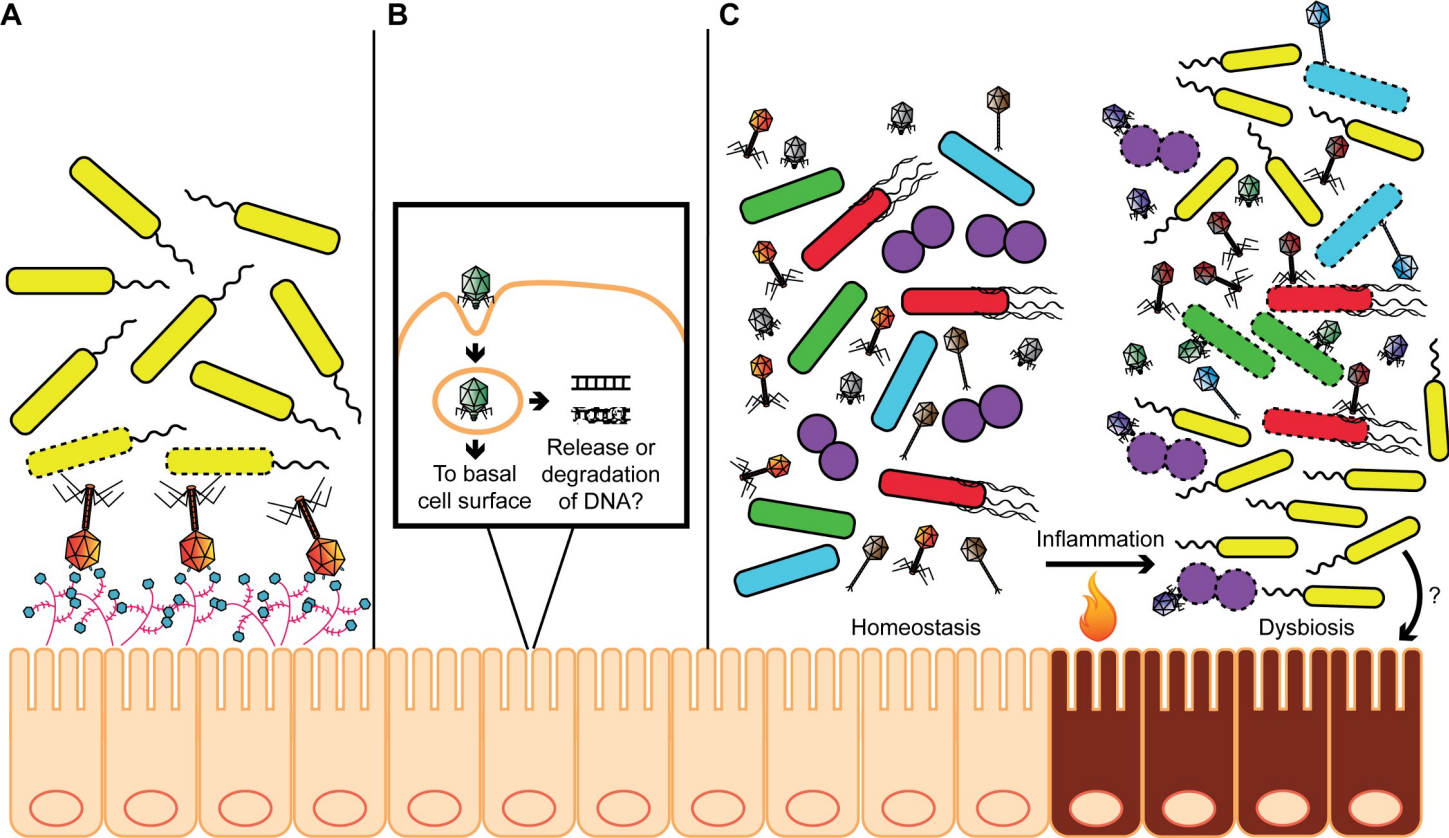
Examples of how phages of the intestinal microbiota could contribute to host–microbe interactions. (A) Phages adhere to mucin glycoproteins at the intestinal mucosal surface using Ig-like domains in their capsids. This adherence, referred to as BAM, is hypothesized to act as a sterilizing barrier against bacteria that invade the mucosa. (B) Bacteriophages can interact with and be internalized into eukaryotic cells. In some instances, phages are exocytosed at the basolateral side of epithelial cells, or they are uncoated and their nucleic acids released inside cells. These nucleic acids either are degraded, have the potential to stimulate the immune system, or contribute to interkingdom gene transfer. (C) Intestinal inflammation experienced during IBD, is correlated with the alteration of phage and bacterial communities to a nonhomeostatic state. During inflammatory stress, phage populations expand—possibly due to enhanced prophage excision—and deplete beneficial members of the bacterial community (indicated by bacterial cells with dashed lines), resulting in the expansion of pathogenic members of the microbiota (yellow rods) that promote disease. Although a direct role of phages during intestinal disease is unknown, they may contribute to bacterial community shifts toward dysbiosis. BAM, bacteriophage adherence to mucin; IBD, inflammatory bowel disease; Ig, immunoglobulin.

The mucosal surfaces of animals are covered with mucin glycoproteins, and phages associate with these surfaces and bind to mucin [7, 19, 20]. Through a mechanism of bacteriophage adherence to mucin (BAM), phages with immunoglobulin (Ig)-like domains in their capsids are capable of binding to host mucins through interactions with glycan moieties [19] (Fig 1A). Ig-like domains are common among phages within the microbiota, and it is hypothesized that BAM provides phage-mediated antibacterial protection of animal mucosal surfaces [19, 21].

Emerging research is beginning to explore interactions between phages and eukaryotic cells. Phages can be endocytosed by epithelial cells and translocated out of cells as intact phage particles [22] (Fig 1B). Phages have been shown to bind to polysialylated glycans that resemble polysaccharide phage receptors on the surface of neuroblastoma cells, resulting in phage internalization and intracellular release of encapsidated phage DNA [23]. Phages associate with mucosal surfaces and can enter and exit eukaryotic cells, and their nucleic acid is uncoated inside cells. This suggests that phages play unidentified roles in *trans*-kingdom interactions such as antimicrobial barrier defense, education of the immune system, and prokaryote–eukaryote DNA transfer (Fig 1A and 1B).

## A role for bacteriophages during disease

The phage component of the intestinal microbiota is an untapped source for further understanding the complexity of host–microbiota interactions during health and disease. Elevated levels of dsDNA phages are associated with inflammatory bowel disease (IBD), and it appears that abundance and diversification of intestinal phages during IBD are independent of changes in the host bacterial community [24, 25]. This suggests that the intestinal immune response during IBD may be a contributing factor to the assembly of phage communities in vivo. Indeed, it has been shown that host inflammation has a strong influence on phage composition in the intestine [26] (Fig 1C). Additionally, alterations in intestinal phage composition precede the development of type 1 diabetes in children [27]. In this study, specific phages were shown to correlate with the onset of disease and suggest that there may be a phage component to the development of autoimmunity. The correlation of aberrant phage community composition to disease status suggests that phages could be utilized as biomarkers for the early detection of diseases in which environmental microbial factors are at play.

## The connection between bacteriophages and the immune system

Renewed interest in phage therapy for the treatment of infectious bacterial diseases has led to paradigm–shifting observations of how phages influence the function of the immune system. A seminal discovery showed that phages collaborate with neutrophils to promote efficient bacterial clearance during lung infection [28]. This process was dependent on functional Toll-like receptor signaling, emphasizing the importance of the immune system in phage-mediated antibacterial activity. Because the lung is an environment rich in natural phage diversity [7], it stands to reason that phages may dictate the health of this environment through interactions with the immune system.

Phages can be important for protecting bacteria from the deleterious effects of the immune system and, in some cases, promote disease. Prophages induced from the *Pseudomonas aeruginosa* chromosome are an important component of in vivo biofilms, a collection of bacteria often residing in polymicrobial communities [29, 30]. These phages stabilize the biofilm by promoting attachment to lung mucus and restrict the dispersal cells from the biofilm, which reduces *P*. *aeruginosa* invasion of epithelial cells [31]. As a component of the biofilm matrix, *P*. *aeruginosa* phages help decrease pro-inflammatory cytokine production and reduce the recruitment of neutrophils to the site of biofilm colonization, thereby perpetuating chronic infections [31].

## Concluding remarks

The study of phage communities within the microbiota and other host-associated polymicrobial environments represents an emerging field, and there are not yet clear criteria for how these resident viruses impact health and disease. Phages are known to influence the metabolic functions of their bacterial hosts and, in this capacity, may indirectly influence mammalian biological processes by altering the physiological state of bacteria. It has been determined that phages strongly influence bacterial community structure and could be harnessed to direct the assembly of bacterial communities to promote microbial homeostasis. Finally, phages are being reconsidered as therapeutic agents to target and kill bacterial pathogens or dysregulated members of the microbiota. The administration of lytic phages or the controlled induction of prophages from bacterial chromosomes may provide new strategies to diversify or restrict microbial communities, likely drawing significant interest towards translational medical applications. The discovery that specific phages are associated with certain diseases suggests that, in addition to therapeutics, phages may serve as relevant biomarkers of disease. The study of phage biology within the context of the mammalian microbiota provides new opportunities to study phage interactions with both bacteria and animals. The future of phage research in biomedicine has great promise and is ripe for new discoveries.
